# Synergistic inhibition of progesterone receptor‐A/B signalling by simvastatin and mifepristone in human uterine leiomyomas

**DOI:** 10.1002/ctm2.1672

**Published:** 2024-04-22

**Authors:** Sadia Afrin, Gregory W. Kirschen, Mariko Miyashita‐Ishiwata, Malak El Sabeh, Mostafa A. Borahay

**Affiliations:** ^1^ Department of Gynecology and Obstetrics The Johns Hopkins Hospital Baltimore Maryland USA; ^2^ Department of Obstetrics and Gynecology Baylor College of Medicine Houston Texas USA

Dear Editor,

We examined the interaction between simvastatin (SIM) and the progesterone pathway for the treatment of fibroids using human tissue and explored cellular mechanisms by which SIM, a hydroxymethylglutaryl‐CoA reductase inhibitor, and mifepristone (MIF), a progesterone receptor (PR) antagonist, act synergistically to inhibit fibroid growth. Using patient‐derived primary leiomyoma cells, immortalized fibroid (human immortalized leiomyoma [HuLM]) cells, and fibroids taken from a xenograft mouse model and patient‐derived surgical specimens in an ongoing phase II randomized controlled trial (RCT) of SIM versus placebo, we investigated mechanistic effects of SIM on PR expression, fibroid cell proliferation, and downstream signalling. Leiomyomas (fibroids) are common benign smooth muscle tumours of the uterus that participate in signalling pathways related to extracellular matrix (ECM) remodelling and cell proliferation.^1^ Leiomyoma cells express PRs whose stimulation increases cellular proliferation.^2^, ^3^ SIM inhibits fibroid growth by impeding proliferation pathways and disrupting mechanotransduction, the cellular sensing of and response to contractile stress.^4^ However, it remains unknown whether SIM acts on these pathways in a PR‐dependent fashion.

To explore whether SIM affects the progesterone system in human leiomyomas in vivo, we immunohistochemically stained pPR‐A/B from patients with leiomyomas enrolled in an ongoing RCT of placebo versus SIM (Figure 1A). We observed suppression of pPR‐A/B expression in all four subjects treated with SIM (Figure 1B,C).

**FIGURE 1:**
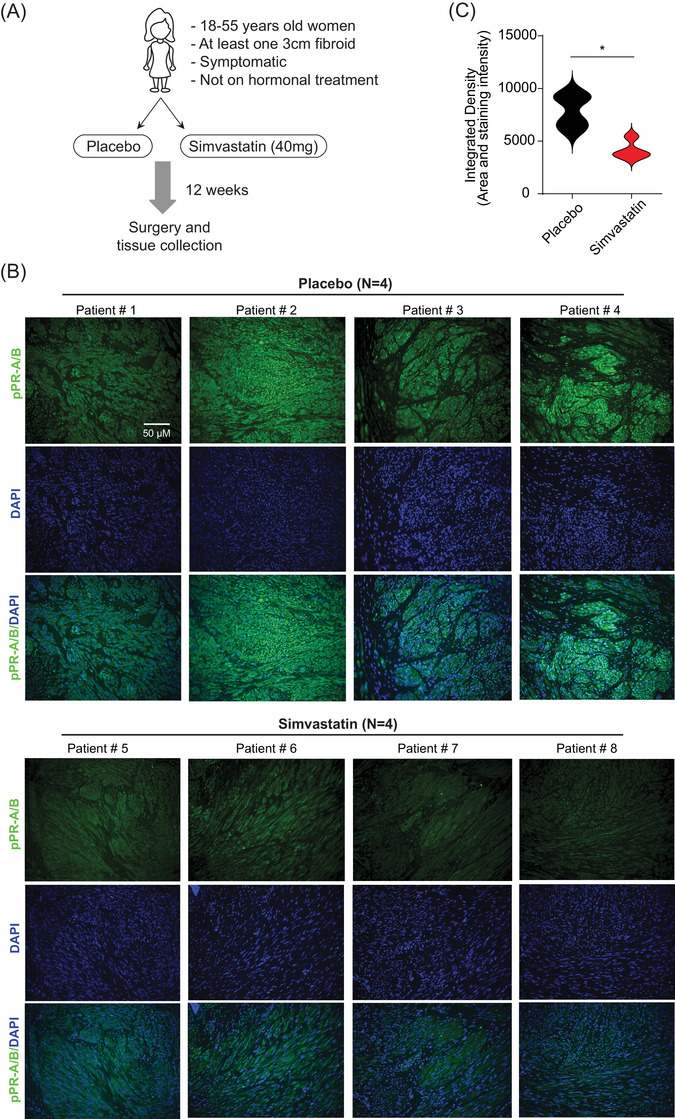
**Suppression of progesterone receptor (PR) expression in simvastatin‐treated clinical tissue**. (A) Diagram of clinical trial design of simvastatin (SIM) versus placebo for treatment of leiomyomas. Patients with uterine fibroids were recruited for treatment with simvastatin (40 mg daily) or a placebo (starch 1500 encapsulated) for a total of 12 weeks. At the end of the treatment, fibroid samples were collected after the surgery to evaluate the effects of simvastatin. (B) Tissue was fixed with a 10% buffered formalin solution for 24 h and kept in 70% ethanol at 4°C. Tissue was then stained with pPR‐A/B antibody (green fluorescence) and 4'‐6‐Diamidino‐2‐phenylindole (DAPI) (blue fluorescence). Scale bars, 50 µm. (C) The optical density was measured and quantified in 15 images using ImageJ, using 10 histologically similar fields randomly selected from each slide. * *p* < .05 versus the placebo.

We next determined whether SIM affects leiomyoma proliferation via a progesterone‐dependent mechanism. We treated primary uterine leiomyoma cells with 100 nM progesterone (P4) and SIM alone or in combination. Using the MTT proliferation assay, we observed significantly elevated proliferation at 48 h compared to vehicle control, with diminished proliferation by 48 h, consistent with prior work^5^, ^6^ (Figure S1A). We treated primary leiomyoma cells with increasing doses of SIM and observed a dose‐dependent decrease in proliferating cell nuclear antigen (PCNA) protein expression, (cellular proliferation marker) (Figure S1B). We performed a patient‐derived xenograft leiomyoma mouse model study of SIM treatment to observe the effects of SIM on PR expression in xenografted mice.^7^, ^8^ Treatment with SIM led to decreased PR expression in vivo (Figure 2A). We corroborated these data with immunofluorescence in human primary leiomyoma cells, noting a similar pattern (Figure 2B). We quantified the expression of PRs A and B (PR‐A/PR‐B) using this paradigm, observing a dose‐dependent decrease in total and phosphorylated PR‐A/PR‐B with increasing SIM concentrations (Figure 2C,D).

**FIGURE 2:**
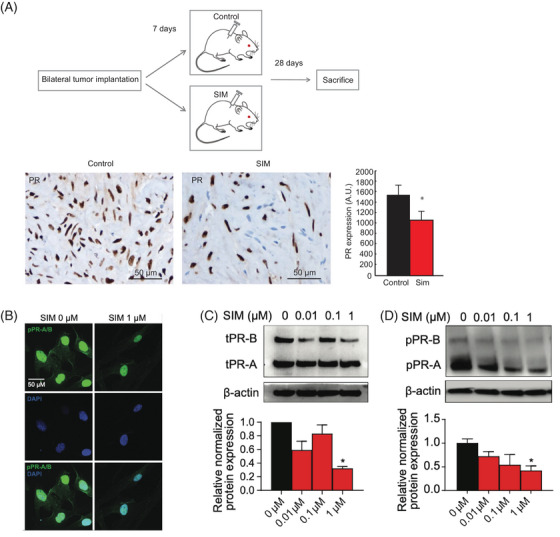
**Simvastatin treatment suppressed progesterone receptor (PR)‐A/B expression in a xenograft mouse model and primary cells**. (A) 6‐week old female immunodeficient NOG (NOD/Shi‐*scid*/IL‐2Rγnull) mice (*n* = 12 control, 11 simvastatin [SIM]) were implanted with two human‐derived fibroid tumours each and assigned to vehicle control or SIM (20 µg/g body weight/day for 28 consecutive days). We stained fibroid tumours for PR and observed significantly decreased expression in the SIM group (*p* = .036). The data presented here are previously unpublished PR expressions from our previously reported xenograft leiomyoma mouse model (cartoon adapted).^7^, ^8^ (B) Immunocytochemistry staining was performed on primary leiomyoma cells to confirm the cellular localization of pPR‐A/B (green fluorescence) and 4'‐6‐Diamidino‐2‐phenylindole (DAPI) (blue fluorescence). (C) total and (D) phosphorated PR‐A/B expression after 48 h SIM treatment (WB). Β‐actin was used as a loading control. Scale bar, 50 µm. Each condition was repeated in triplicate. Each graph represents the experimental data with standard error of the mean (SEM) of three independent experiments with similar results. *, *p*  <  .05 versus the control; #, *p*  <  .05 versus P4.

To investigate the downstream signalling of SIM on primary leiomyoma cells, we performed Western blots for key proliferation‐related proteins in SIM‐treated cells. We observed a dose‐dependent decrease in pERK1/2/ERK1/2, pJNK1/2/JNK1/2, pAKT/AKT and pmTOR1/mTOR1 with increasing SIM doses (Figure 3A–D). We noted decreased PGMCR1, a PR interacting partner, with increasing concentrations of SIM (Figure 3E). SIM downregulated PR in leiomyoma cells in a dose‐dependent fashion using a luciferase reporter assay (Figure 3F).

**FIGURE 3:**
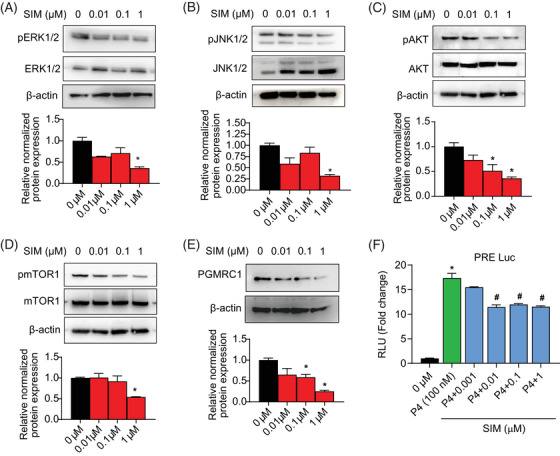
**Inhibition of progesterone downstream signalling and transcription by simvastatin**. The expression of progesterone receptor (PR) downstream signalling (A) pERK/ERK, (B) p‐JNK/JNK, (C) pAKT/AKT, (D) pmTOR1/mTOR1 and (E) PGMRC1 were assessed in primary leiomyoma cells after simvastatin (SIM) treatment. Protein expressions were assessed using the Western blot (WB) assay. β‐actin was used as a loading control. Dimethyl sulfoxide (DMSO) was used as vehicle control. (F) Transcriptional activity of progesterone in human immortalized leiomyoma (HuLM) cells after simvastatin treatment. Leiomyoma cells were co‐transfected with plasmids of PRE‐luc (500 ng/well) and PR‐B (50 ng/well) for 8 h. Cells were treated with P4 (100 nM) and SIM (0.001–10 µM) alone and combination for 48 h. Cells were harvested and assayed for luciferase activity. The data is presented as relative light units (RLU) defined as the luciferase output normalized to protein content. The fold changes were calculated by normalization with luciferase value with no P4 treatment. Each condition was repeated in triplicate. Each graph represents the experimental data with SEM of three independent experiments with similar results. *, *p*  <  .05 versus the control; #, *p*  <  .05 versus P4.

Leiomyoma cells interact with their surrounding ECM.^4^, ^9^ Specifically, they overexpress Integrin β1 and participate in mechanotransduction signalling.^9^ SIM decreases the expression of integrin β1 and downstream signalling via focal adhesion kinase (FAK), leading to increased cellular contractility and increased ECM stiffness in primary leiomyoma cells.^4^ To determine whether SIM affects the leiomyoma mechanotransduction not only at the level of intracellular FAK but also via extracellular proteins, we measured levels of proteins important in ECM and mechanotransduction dynamics in primary leiomyoma cells treated with SIM. Consistently, we found that SIM dose‐dependently downregulated collagen 1A, versican, and fibronectin (Figure S2A–C).

To probe the interaction between SIM and PR pathway, we co‐incubated P4‐treated leiomyoma cells with SIM and a PR blocker, MIF and examined proliferation via PCNA expression. P4 alone trended toward increasing PCNA in cultured primary leiomyoma cells (Figure 4A). By contrast, SIM alone and MIF alone decreased PCNA levels (Figure 4A). In P4‐pretreated cells treated with both SIM and MIF, we observed a synergistic effect, with lower levels of PCNA than with either alone (Figure 4A). This suggests that SIM acts in parallel with MIF to inhibit P4‐induced leiomyoma cell proliferation. We verified that downstream signalling partners of the PR pathway, ERK1/2, JNK1/2 and pAKT were synergistically downregulated by the combination of SIM and MIF in P4‐treated cells (Figure 4B–D). Conversely, we were curious whether overexpression of PR (as opposed to its inhibition by MIF) would impact SIM's effect on leiomyoma proliferation. We cultured HuLM cells co‐transfected with Pre‐luc reporter and PR‐B, then treated cells with P4, SIM, and the two in combination, observing significantly elevated PCNA expression in PR‐B‐overexpressed cells treated with P4, while SIM inhibited this expression both in overexpressed cells without P4 and with P4 (although to a lesser extent in presence of P4) (Figure S3).

**FIGURE 4:**
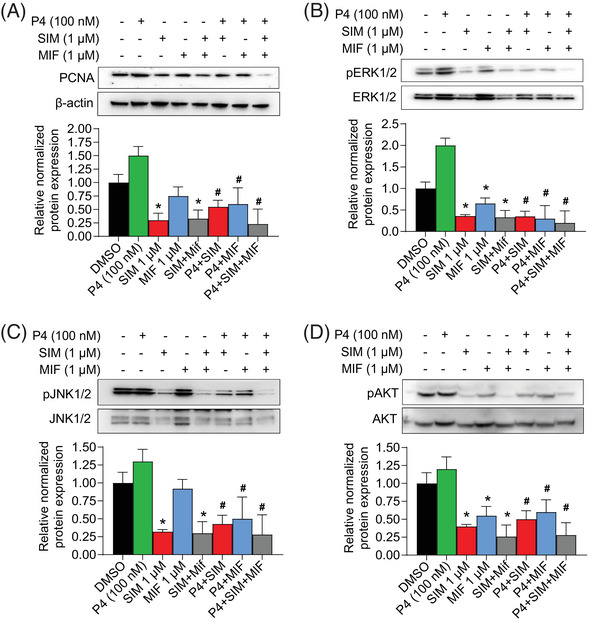
**Simvastatin and mifepristone synergistically inhibit proliferation and downstream targets**. The expressions of (A) PCNA, (B) pERK1/2/ ERK1/2, (C) pJNK1/2/ JNK1/2 and (D) pAKT/ AKT were assessed in primary leiomyoma cells after treatment with P4 (100 nM), simvastatin (SIM) (1 µM), Mifepristone (MIF,1 µM) alone or combination for 48 h. Protein expressions were assessed using WB assay. β‐actin was used as a loading control. Dimethyl sulfoxide (DMSO) was used as vehicle control. Each condition was repeated in triplicate. Each graph represents the experimental data with SEM of three independent experiments with similar results. *, *p*  <  .05 versus the control; #, *p*  <  .05 versus P4.

We finally sought to determine whether SIM and MIF synergistic inhibition would manifest in the mechanotransduction pathway. We performed an analogous experiment to that described in Figure 4. In P4‐pretreated primary leiomyoma cells, co‐treatment with SIM and MIF led to synergistic downregulation of ECM‐associated proteins collagen 1A and fibronectin (Figure S4A,B). Likewise, the integrin pathway was synergistically downregulated by SIM and MIF co‐treatment, as exemplified by decreased levels of Integrin β1 and downstream target pFAK (Figure S4C,D). This suggests that SIM interacts with the PR pathway to affect ECM protein dynamics and mechanotransduction.

## 1 CONCLUSION

Here, we explored a novel mechanism whereby SIM exerts anti‐fibroid effects in human tissue by targeting the PR and downstream signalling partners. This anti‐tumor effect of SIM (albeit modest, reducing cell viability by ∼25%) was shown in cell culture and in xenograft mouse model. This modest effect may be due to the slow proliferation of fibroid tumours. Further, we noted reduction in cell viability between the control and SIM 0.01 µM is significant, whereas we found a non‐significant difference in PCNA expression between these two groups. Differences in what these two assays measure may explain the discrepancy: the MTT assay measures cellular metabolism, whereas PCNA expression measures DNA replication. While P4 trended towards inducing proliferation over vehicle control, this was not statistically significant, therefore SIM and MIF effects on fibroid tissue are likely multifactorial including non‐proliferative mechanisms. Figure S5 shows a diagram of the multi‐pronged targeting of fibroids by SIM and MIF.

## AUTHOR CONTRIBUTIONS

Conceptualization, SA and MAB; methodology, SA and MAB; investigation, SA, MM‐I, MEL and MAB; analysis, SA, GWK and MAB; writing and editing: SA, GWK and MAB; supervision and funding, MAB.

## CONFLICT OF INTEREST STATEMENT

The authors declare no conflict of interest.

## FUNDING INFORMATION

This research was funded by NIH grant number R01HD094380 to Mostafa A. Borahay and the Kelly Society to Gregory W Kirschen.

## ETHICS STATEMENT

The Institutional Review Board of Johns Hopkins University reviewed and approved the study (IRB00196175). Written informed consent was obtained from all subjects involved in the study.

## Supporting information

Supporting Information

Supporting Information

Supporting Information

Supporting Information

Supporting Information

## Data Availability

Data are available upon request to the corresponding author.
